# RNAi Therapeutics in Autoimmune Disease

**DOI:** 10.3390/ph6030287

**Published:** 2013-03-05

**Authors:** Kaleb M. Pauley, Seunghee Cha

**Affiliations:** 1 Department of Science and Mathematics, Cedarville University, 251 North Main Street, Cedarville, OH 45314, USA; E-Mail: kpauley@cedarville.edu; 2 Department of Oral and Maxillofacial Diagnostic Sciences, University of Florida College of Dentistry, 1600 SW Archer Rd, Gainesville, FL 32610, USA

**Keywords:** RNA interference, therapeutics, microRNA, small interfering RNA, autoimmune disease

## Abstract

Since the discovery of RNA interference (RNAi), excitement has grown over its potential therapeutic uses. Targeting RNAi pathways provides a powerful tool to change biological processes post-transcriptionally in various health conditions such as cancer or autoimmune diseases. Optimum design of shRNA, siRNA, and miRNA enhances stability and specificity of RNAi-based approaches whereas it has to reduce or prevent undesirable immune responses or off-target effects. Recent advances in understanding pathogenesis of autoimmune diseases have allowed application of these tools *in vitro* as well as *in vivo* with some degree of success. Further research on the design and delivery of effectors of RNAi pathway and underlying molecular basis of RNAi would warrant practical use of RNAi-based therapeutics in human applications. This review will focus on the approaches used for current therapeutics and their applications in autoimmune diseases, including rheumatoid arthritis and Sjögren’s syndrome.

## 1. Introduction

RNA interference (RNAi) is a cellular mechanism occurring in most eukaryotic cells that is responsible for post-transcriptional gene regulation. This process involves small single stranded RNA molecules binding to the 3' UTR of specific mRNA targets resulting in the degradation or translational repression of that target. Since the discovery of the RNAi pathway, there has been a focused interest on the development of RNAi-based therapeutics for otherwise “undruggable” targets. This review will outline the mechanism of RNAi and approaches to RNAi-based therapeutic strategies and address the progress of RNAi-therapeutics for autoimmune diseases.

### The Effectors of RNA Interference

The effectors of the RNAi pathway are small RNA (RNA) molecules that can be classified into at least three categories based on their origin and function: microRNAs (miRNA), short interfering RNAs (siRNA), and short hairpin RNAs (shRNA).

MiRNAs represent endogenously encoded small RNAs, about 22 nucleotides long, which function to post-transcriptionally repress gene expression by binding to target mRNAs in a sequence specific manner. Though discovered relatively recently, miRNA are now recognized as key regulators of gene expression in plants and animals. The genes encoding miRNAs are transcribed by RNA polymerase II in a long primary miRNA that is cleaved in the nucleus by Drosha/DGCR8 into a precursor miRNA (pre-miRNA). The pre-miRNA consists of about 70 base pairs with a single-stranded hairpin loop. Pre-miRNA is exported from the nucleus via exportin-5, and is cleaved by Dicer in the cytoplasm to generate a mature miRNA about 22 nucleotides long. This mature miRNA is incorporated into a complex known as the RNA-induced silencing complex (RISC) where it binds to its target mRNA though complementary base pairing. If complementarity is perfect, mRNA degradation typically occurs, whereas if complementarity is partial, translational repression typically occurs [1].

The shRNAs are dsRNAs engineered with a similar structure as miRNA, and are either tranfected into cells directly or a vector encoding the shRNA is introduced into the cells and transcribed.

SiRNA molecules are synthetic dsRNAs ranging in size from about 19–30 nucleotides that function to cause mRNA degradation through the perfect pairing of complementary sequences [2]. These molecules have become invaluable research tools to study the functions of genes and an alternative to knock-out mice.

## 2. Approaches to RNAi Therapeutics

### 2.1. Short Hairpin RNAs

One advantage of using shRNAs is that they assimilate into the endogenous RNAi pathway, and are therefore more efficient than exogenously introduced siRNAs [3]. Using a viral vector-based delivery system for shRNAs increases transfection efficiency and allows harder-to-transfect cells such as non-dividing neuronal cells to be targeted. However, there are obvious safety concerns associated with the use of viral expression vectors in humans. Other delivery strategies for shRNA include coupling with monoclonal antibodies, peptides, or aptamers that recognize specific cell surface molecules, but each of these strategies are associated with increased toxicity [4,5,6,7].

### 2.2. MicroRNAs

In recent years, differential expression of miRNA has been associated with a wide variety of human diseases ranging from many types of cancer to autoimmune diseases and everything in between. This revelation makes them an attractive target for therapies. The premise is that if we can elucidate reliable techniques to alter miRNA levels in specific cells/tissues, perhaps we can ameliorate disease symptoms or cure diseases altogether. There are two main strategies that can be employed to do this. The first is the use of synthetic miRNA mimics to restore the expression level of a particular miRNA back to normal levels [8]. This strategy presents many of the same challenges faced by siRNA therapies to be discussed later, such as stability and delivery. In addition, most miRNAs are pleiotropic hence altering levels of any given miRNA may result in unintended consequences. The second strategy is the use of miRNA “sponges” to block the action of over-expressed miRNAs [8]. Again, many of the same challenges apply to the use of sponges including design, delivery, and the pleiotropic nature of most miRNAs.

### 2.3. Small Interfering RNAs

#### 2.3.1. Design

The main focus of this review will be on the use of siRNA molecules as therapeutic agents. When considering the use of siRNA for therapeutic purposes, the first step is selection of an appropriately designed siRNA that takes into account potency, specificity and stability. Algorithms have been developed to assist in designing potent siRNA based on the target gene of interest, but once synthesized, these siRNAs must be experimentally evaluated to determine their efficacy in gene silencing. Specificity is another critical factor to consider. Although it has been demonstrated that siRNAs can have selective silencing of genes that differ by a single nucleotide in sequence [9], siRNAs can have “off-target” effects where expression of homologous mRNAs is altered or unrelated genes are altered [10]. Furthermore, siRNAs may induce the innate immune response by activating Toll-like receptors such as TLR7 that recognizes viral dsRNA. This could lead to a potentially harmful inflammatory response in patients.

#### 2.3.2. Stability

When considering the use of siRNA molecules in *in vivo* models, stability of the molecule becomes another critical factor. Unprotected siRNA molecules are highly unstable and easily degraded in human plasma, with a half-life of only five minutes [11,12]. Chemical modifications are necessary to extend the half-life of siRNA *in vivo* without altering efficacy of gene silencing. Such chemical modifications include alterations in the phosphate groups linking nucleotides that confer exonuclease resistance or alterations (typically methylation) in the sugar residues that confer endonuclease resistance. The right balance between increased stability and maintained efficacy must be experimentally determined for siRNA molecules.

#### 2.3.3. Delivery

Delivery of siRNA molecules to target cells/tissues is perhaps the most challenging factor of RNAi therapeutics. Due to its negative charge, siRNA molecules do not penetrate cell membranes easily. Only a few anatomically isolated sites have been shown to exhibit gene silencing after uptake of naked siRNA. This opens up the possibility for siRNA use in these areas (eyes, lungs, central nervous system), but for more systemic use, delivery strategies must be developed. siRNA delivery can be achieved by a variety of strategies including lipid-based formulations [13], nanoparticles [14], and magnetofection [15]. However, these strategies are nonspecific, and cell-type specific delivery is still the most challenging step blocking the progress of RNAi therapy in modern medicine. In order to target siRNA to specific cell or tissue types, specificity must be built into the delivery agents or expressed shRNAs. Some strategies for cell-type specific delivery include antibody targeting [16], cell-penetrating peptides [17], chemical modifications [13], and aptamers [7], but each of these strategies presents certain drawbacks such as cytotoxicity or immunogenicity.

Recently, Lima *et al.* demonstrated that single-stranded siRNA molecules are capable of functioning through the RNAi pathway in animals and result in potent gene silencing [18]. This finding is significant, because single-stranded siRNAs offer several advantages over dsRNAs. The lack of a second “passenger” strand reduces the risk for off-target effects, increasing specificity of the siRNA. More importantly, ssRNAs are easily delivered *in vivo* without any lipid-based transfection reagents or other delivery vectors. Chemically modified, naked single-stranded siRNAs can be delivered *in vivo* using a simple saline solution and result in potent gene silencing. This strategy may prove very useful for a systemic therapeutic approach using siRNA. However, in many diseases, specific cell-types or tissues must be targeted, thus a more specific delivery method must be developed.

## 3. RNAi Therapeutics for Autoimmune Diseases

The discovery of RNAi as a sequence-specific mechanism of gene regulation has made it an attractive concept for the development of new therapies. Numerous clinical trials for RNAi therapeutics have been completed or are ongoing for various diseases such as age-related macular degeneration (AMD), glaucoma, respiratory syncytial virus (RSV), asthma, liver cancer, and pancreatic cancer (clinical trials.gov). This review will focus on the progress of RNAi therapeutic development in autoimmune diseases.

### 3.1. *In Vitro* Studies

One study set out to develop a siRNA-based therapeutic targeting Sjögren’s syndrome (SjS), an autoimmune disease characterized by lymphocytic infiltration in the salivary and lacrimal glands resulting is severe dry mouth and dry eye symptoms due in part to apoptotic cell death of secretory acinar cells in the glands [19]. Currently, the only course of treatment available to SjS patients is immunosuppressive therapy and secretagogues, a class of drugs that stimulate secretion. Pauley *et al.* designed a conjugate composed of a siRNA molecule targeting caspase-3 and a muscarinic receptor agonist, carbachol [19]. Carbachol binds to muscarinic receptors inducing secretion and is endocytosed via receptor-mediated endocytosis. Linking a siRNA molecule to carbachol allows cellular uptake of the siRNA through this receptor-mediated endocytic pathway and specific delivery of the siRNA since only cells expressing muscarinic receptors including salivary acinar cells would be targeted. Pauley *et al.* showed that both the siRNA and carbachol remained functional after conjugation and HSG cells treated with conjugate (in the absence of transfection reagent) exhibited significant reduction in caspase-3 gene/protein expression, indicating that the conjugate is taken up by cells via muscarinic receptor-endocytosis. More importantly, inflammatory cytokine-induced apoptosis of HSG cells was inhibited by conjugate treatment [19]. These data suggest that this strategy may be useful to prevent apoptotic cell death of secretory acinar cells in SjS patients, thus maintaining secretory function to improve quality of life for the patients. Further studies are needed to continue developing this conjugate for *in vivo* use.

### 3.2. *In Vivo* Studies

Several studies have examined the potential use of siRNA therapy in rheumatoid arthritis (RA). In 2005, Schiffelers *et al.* investigated the use of localized electroporation for *in vivo* delivery of siRNA into joint tissue of arthritic mice [20]. It was demonstrated that electroporation of anti-TNFα siRNA resulted in inhibition of joint inflammation in collagen-induced arthritis. A later study by Khoury *et al.* in 2006 utilized a DNA molecule for complexation and a cationic liposome for the intravenous delivery of anti-TNFα siRNA in collagen-induced arthritic mice [21]. When administered weekly, a 50%–70% reduction of TNF-α and an almost complete ablation of arthritic symptoms was observed in siRNA-treated mice. However, this systemic delivery approach resulted in uptake of siRNA by multiple organs including brain, lungs, liver and spleen.

A more recent study extended this work by testing the efficacy of a “wraposome” to systemically deliver siRNA to arthritic joints in collagen-induced arthritic mice [22]. A wraposome consists of a core composed of a cationic lipid bilayer and siRNA complex enveloped in a neutrak lipid bilayer with polyethylene glycol on the surface. This design results in a neutral surface charge and prevents the non-specific binding of the liposome to plasma proteins after intravenous injection. Collagen-induced arthritic mice receiving siRNA targeting TNF-α via this systemic wraposome delivery exhibited significant decreases in the severity of arthritis coupled with reduction of TNF-α in the joints. Interestingly, the siRNA was mainly incorporated into macrophages and neutrophils in the inflamed synovium [22]. These findings support the potential therapeutic effects of suppressing inflammatory molecules via siRNA in rheumatoid arthritis.

Yet another study examining the potential use of RNAi therapy in an arthritis animal model used a different strategy. Chen *et al.* used a lenti-viral vector to deliver short hairpin RNA targeting TLR7 in collagen-induced arthritic mice [23]. Again, severity of arthritis symptoms and expression of inflammatory mediators in the joints were reduced in treated mice compared to untreated mice.

In addition to siRNA therapeutic potential in RA, certain miRNAs, particularly miR-146a, have been found to be differentially expressed in RA and other autoimmune diseases such as Sjögren's syndrome and systemic lupus erythematosus (SLE) [24,25,26,27,28,29,30,31,32]. One study demonstrated the potential therapeutic application of miR-146a for RA by demonstrating the inhibitory effect of miR-146a on osteoclastogenesis *in vitro* [33]. Furthermore, Nakasa *et al.* were able to demonstrate that intravenous administration of miR-146a resulted in the suppression of cartilage and bone destruction in a collagen-induced arthritis mouse model [33]. These findings support the therapeutic potential of miRNA modulation in autoimmune diseases. However, further studies are needed to identify and understand differentially expressed miRNAs in autoimmune disease patients.

## 4. Conclusions

Despite significant progress in the field of RNAi therapeutics, there is still much research needed to perfect the design and delivery of siRNA in humans. Although several clinical trials using RNAi therapeutics have been completed, new problems have surfaced including unintended immunological effects, effects on the endogenous RNA silencing pathway, and the ultimate dilemma of how to effectively deliver siRNA *in vivo*. Regardless of these setbacks, hope still remains that with time, RNAi therapeutics will prove to be the next “superdrug” against a variety of human diseases.
